# Prolonged preoperative double J stenting increases post-ureteroscopy infectious complications

**DOI:** 10.1007/s00345-025-06042-0

**Published:** 2025-10-29

**Authors:** Matteo Ortolini, Beatrice Breu, Audrey Masnada, François Crettenand, Kevin Stritt

**Affiliations:** Department of Urology, University Hospital Lausanne (CHUV), University of Lausanne, Rue du Bugnon 46, 1011 Lausanne, Switzerland

**Keywords:** Nephrolithiasis, Double J stenting, Infectious complications

## Abstract

**Background:**

The clinical benefit of preoperative ureteric double J (JJ) stenting prior to ureterorenoscopy (URS) for uncomplicated urolithiasis remains debated. In cases requiring urgent decompression or delayed definitive treatment, JJ stenting is frequently employed. However, prolonged indwelling time may increase the risk of bacterial colonization and subsequent infectious complications, though evidence remains limited.

**Methods:**

We conducted a retrospective, single-center study including 350 adult patients who underwent URS at the Department of Urology, Lausanne University Hospital (CHUV) between January and December 2023. The primary outcome was infectious complication, defined as the occurrence of ≥ 1 of the following within 30 days postoperatively: fever > 38.0 °C, systemic inflammatory response, hospitalization > 3 days with documented signs of infection, or readmission for urinary infection. Ten predefined clinical variables were analyzed using univariable and multivariable logistic regression to identify independent predictors of infectious failure.

**Results:**

Most patients (83%) had a stent in place at the time of surgery, and 78% received cefuroxime as prophylaxis. Infectious complications occurred in 29 patients (8.3%). Patients with infectious complications had significantly longer JJ stent dwell times (mean 63.9 vs. 36.3 days, *p* < 0.001). Multivariable analysis identified prolonged stent dwell time (OR 0.984 per day; 95% CI 0.973–0.995; *p* < 0.001) and neurogenic bladder (OR 0.871; 95% CI 2.196–6.739; *p* < 0.001) as independent risk factors for infectious failure. Subgroup analysis revealed a significant increase in infection rates when dwell time exceeded 60 days (*p* < 0.001).

**Conclusion:**

Prolonged JJ stent dwell time and neurogenic bladder are independently associated with increased postoperative infectious complications after URS. Our findings support implementing fast-track surgical protocols to reduce stent duration, particularly avoiding delays beyond 60 days, to minimize infection-related morbidity.

## Introduction

Urolithiasis is a common and increasingly prevalent condition worldwide. In the United States, the estimated lifetime prevalence of kidney stones is approximately 12% in men and 10% in women [1–3]. Beyond being a frequent cause of acute flank pain and emergency department visits, kidney stones are associated with substantial healthcare costs, high recurrence rates, and a significant decline in patient quality of life [4–6]. Despite the availability of effective treatment options, long-term management remains challenging due to frequent recurrences.

When surgical intervention is warranted, ureterorenoscopy (URS) is a widely adopted and effective modality for stone removal. In many cases—particularly in the setting of obstructive uropathy or infection—temporary ureteric double J (JJ) stenting is employed preoperatively to decompress the urinary tract and facilitate access during URS. However, procedural delays due to logistical or institutional constraints can lead to prolonged stent indwelling, which may increase the risk of bacterial colonization, urinary tract infection, and postoperative infectious complications [7–10]. Notably, preoperative JJ stent dwell times exceeding two months have been independently associated with a significantly higher incidence of febrile urinary tract infections after URS [7, 8]. These findings emphasize the need to minimize stent duration as part of an optimized strategy to reduce infection-related morbidity in endourological practice.

The objective of this study was to evaluate whether prolonged preoperative JJ stenting is independently associated with an increased risk of postoperative infectious complications following URS. Identifying this potential risk factor could support the implementation of fast-track surgical pathways aimed at reducing unnecessary stent dwell time and improving clinical outcomes.

## Materials and methods

We conducted a retrospective, single-center study in the Department of Urology at Lausanne University Hospital (CHUV), Switzerland. It included all patients who underwent URS between January 1 and December 31, 2023. Data were extracted from the hospital’s secure electronic medical records system (Soarian^®^ Clinicals, Cerner Corporation, Kansas City, MO, USA), which records demographic, clinical, laboratory, radiological, and operative data as part of routine care. No additional data or biological samples were collected for this study.

The study population consisted of adult patients who underwent URS, regardless of sex or age. Preoperative JJ stents were placed based on clinical judgment, primarily for obstructing ureteral stones, with or without infection. Only patients with complete medical records documenting preoperative urine culture results, duration of JJ stenting, intraoperative parameters, and postoperative follow-up were included. Patients with calculi at any location within the ureter or kidney were considered eligible, provided they were legally competent adults at the time of the intervention. Exclusion criteria included deceased individuals, patients who explicitly refused the use of their data for research, and cases lacking diagnostic certainty. No formal power analysis was performed, but the sample size of 350 patients was sufficient to detect significant differences in infectious outcomes.

URS were performed using semi-rigid or flexible ureteroscopes, depending on stone location. A lithoclast device was used for stone fragmentation during rigid URS, whereas a thulium laser (power range 0.8–1.5 J, frequency 8–30 Hz) was used for flexible URS. The use of ureteral access sheaths (UAS) was at the discretion of the surgeon based on ureteral anatomy and stone characteristics.

The primary outcome was defined as infectious complication within 30 days post-URS, corresponding to the occurrence of one or more of the following: fever > 38.0 °C, systemic inflammatory response, hospitalization > 3 days with documented signs of infection, or readmission for urinary tract infection. We assessed the association between infectious failure and ten predefined clinical variables: age, sex, body mass index (BMI), American Society of Anesthesiologists (ASA) score, presence of urinary tract anomalies, neurogenic bladder, indication (urolithiasis vs. malignancy), preoperative positive urine culture, operative time, and duration of preoperative JJ stenting (dwell time).

Statistical analysis included descriptive statistics to summarize patient demographics, clinical characteristics, and complication rates. Comparative analyses were conducted using Chi-square tests for categorical variables and Student’s *t* tests or nonparametric equivalents for continuous variables, depending on data distribution. Univariable and multivariable logistic regression models were then conducted to identify independent predictors of infectious complications. All regression analyses were performed using Stata version 17.0 (StataCorp LLC, College Station, TX, USA). A two-sided *p* value < 0.05 was considered statistically significant. To assess the impact of stent dwell time on infectious outcomes, patients were stratified into three groups based on dwell duration: <30 days, 31–60 days, and > 60 days. Group comparisons were performed using contingency tables with chi-square tests to evaluate differences in infection rates across categories.

### Ethics approval and consent to participate

This retrospective study was approved by the Commission cantonale d’éthique de la recherche sur l’être humain du Canton de Vaud (CER-VD), Lausanne), in accordance with institutional and cantonal guidelines. Data were pseudonymized to protect participant confidentiality. General consent for research use of data has been systematically collected since 2020. For individuals who were lost to follow-up, deceased, or unreachable, a waiver of consent was obtained under Article 34 of the Cantonal Law on Health (LRH). No data were used from patients who explicitly declined the use of their medical information.

## Results

A total of 350 patients who underwent URS during the study period were included in the analysis. The median age was 56 years (IQR 44–69), and 244 patients (70%) were male. The median body mass index (BMI) was 26.8 kg/m² (IQR 23.8–30.5). Regarding ASA classification, 42 patients (12%) were ASA I, 219 (63%) ASA II, 83 (24%) ASA III, and 6 (2%) ASA IV. Urological comorbidities were present in several patients, with 23 individuals (7%) having urinary tract anomalies. These included ureteropelvic junction (UPJ) obstruction in 5 cases (22%), ureteral malformation in 10 cases (43%), cystectomy with urinary diversion in 2 cases (9%), kidney transplant in 3 cases (13%), and other causes in 3 cases (13%). Eight patients (2%) had neurogenic bladder, six of whom (75%) required bladder catheterization. The primary indication for URS was urolithiasis, accounting for 316 patients (90%), of whom 178 (56%) presented with a first episode and 138 (44%) with recurrent stones. Urogenital malignancy was the indication in 34 cases (10%). Baseline demographic characteristics, urological comorbidities, and indications for URS are summarized in Table 1.

**Table 1:** Baseline demographic, urological comorbidities, and indications for URS

Characteristic	Total (*N* = 350)
Median age—year (IQR)	56 (44–69)
Sex, male—no. (%)	244 (70)
Sex, female—no. (%)	106 (30)
Median BMI—kg/m² (IQR)	26.8 (23.8–30.5)
ASA score—no. (%)	
1	42 (12)
2	219 (63)
3	83 (24)
4	6 (2)
Urological comorbidities	
Urinary tract anomalies—no. (%)	23 (7)
UPJ obstruction	5/23 (22)
Ureteral malformation	10/23 (43)
Cystectomy with diversion	2/23 (9)
Kidney transplant	3/23 (13)
Other	3/23 (13)
Neurogenic bladder—no. (%)	8 (2)
With bladder catheterization—no. (%)	6/8 (75)
Indications for URS—no. (%)	
Urolithiasis	316 (90)
First episode	178/316 (56)
Recurrence	138/216 (44)
Urogenital cancer	34 (10)

Data are presented as number (percentage) or median (interquartile range, IQR), as appropriate

*BMI* body mass index, *ASA* American Society of Anesthesiologists score, *UPJ* ureteropelvic junction, *URS* ureterorenoscopy

**Fig. 1:**
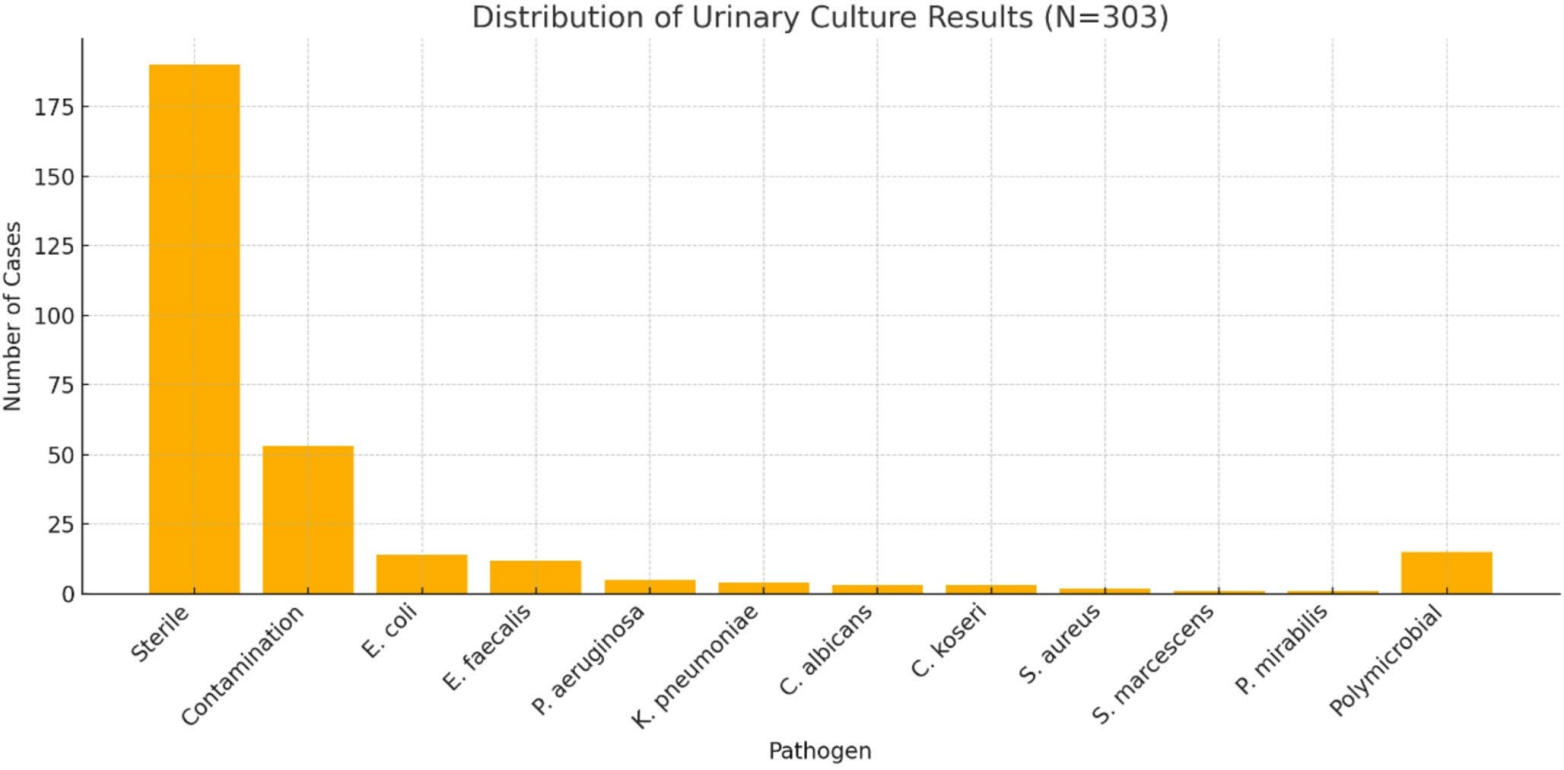
Distribution of urinary culture results among patients with available preoperative cultures (*N* = 303)

Preoperative urinary culture was available for 303 patients (87%) (Table 2; Fig. 1). Among these, 190 (63%) had sterile urine, and 53 (17%) showed contamination. The most frequently identified pathogens were Escherichia coli (5%) and Enterococcus faecalis (4%), followed by Pseudomonas aeruginosa (2%). The median time from culture to URS was 7 days (IQR 7–11).

**Table 2:** Urinary culture results

Urinary culture	Total (*N* = 350)
Pathogen identified:	
Sterile culture	190 (63)
Contamination	53 (17)
*Escherichia coli*	14 (5)
*Enterococcus faecalis*	12 (4)
*Pseudomonas aeruginosa*	5 (2)
Klebsiella pneumoniae group	4 (1)
*Candida albicans*	3 (1)
*Citrobacter koseri*	3 (1)
*Staphylococcus aureus*	2 (1)
Serratia marcescens group	1 (0)
Proteus mirabilis	1 (0)
Polymicrobial culture	15 (5)
Median time from culture to URS-days (IQR)	7 (7–11)

Data are presented as number (percentage) or median (interquartile range, IQR), as appropriate. Contamination refers to mixed flora or non-uropathogenic isolates not meeting criteria for true infection. “Polymicrobial culture” includes samples with two or more distinct uropathogens

*URS* ureterorenoscopy

Regarding surgical approach, rigid URS was performed in 133 patients (38%), flexible URS in 126 (36%), and a combined rigid and flexible procedure in 91 patients (26%). At the time of URS, a JJ stent was in place in 289 cases (83%), while 61 patients (17%) underwent the procedure without prior stenting.

Antibiotic prophylaxis was administered in accordance with institutional guidelines. The most used agents were cefuroxime in 272 patients (78%), followed by amoxicillin/clavulanic acid (7%), ceftriaxone (5%), and other less frequently used antibiotics including sulfamethoxazole/trimethoprim, piperacillin-tazobactam, ciprofloxacin, gentamycin, clindamycin, ertapenem, vancomycin, fluconazole, amikacin, and norfloxacin. The median operative time was 44 min (IQR 31–62) (Table 3).

**Table 3:** Interventions

Intervention details	Total (*N* = 350)
URS approach—no. (%)	
Rigid	133 (38)
Flexible	126 (36)
Rigid + flexible	91 (26)
JJ Stent in situ—no. (%)	
Yes	289 (83)
No	61 (17)
Antibiotic prophylaxis—no. (%)	
Cefuroxime	272 (78)
Amoxicillin/clavulanic acid	26 (7)
Ceftriaxone	16 (5)
Sulfamethoxazole/trimethoprim	7 (2)
Piperacillin-tazobactam	7 (2)
Ciprofloxacin	6 (2)
Gentamycin	5 (1)
Clindamycin	3 (1)
Ertapenem	2 (1)
Vancomycin	2 (1)
Fluconazole	2 (1)
Amikacin	1 (0)
Norfloxacin	1 (0)
Median operative time—min (IQR)	44 (31–62)

Data are presented as number (percentage) or median (interquartile range, IQR), as appropriate. Antibiotic prophylaxis was administered per institutional protocols; agents listed reflect initial perioperative dosing

*URS* ureterorenoscopy, *JJ stent* double-J ureteral stent

Postoperative infectious complications occurred in 29 patients (8.3%) (Table 4). Among these, 7 patients (2%) experienced fever > 38.0°°C, 8 patients (2%) showed systemic inflammatory response, 22 patients (6%) required hospitalization for more than 3 days, and 9 patients (3%) were readmitted within 30 days due to infection.

**Table 4:** Infectious complications

Infectious complications	Total (*N* = 350)
Fever > 38.0 °C—no. (%)	7 (2)
Systemic inflammatory response—no. (%)	8 (2)
Hospitalization > 3 days—no. (%)	22 (6)
Readmission within 30 days for infection—no. (%)	9 (3)

Data are presented as number (percentage)

Univariable and multivariable logistic regression analyses were performed to identify predictors of infectious complication. In multivariable analysis, prolonged JJ stent dwell time was independently associated with an increased risk of infection (OR 0.984 per day; 95% CI 0.973–0.995; *p* < 0.001), as was the presence of a neurogenic bladder (OR 0.871; 95% CI 2.196–6.739; *p* < 0.001). Other variables, including age, BMI, ASA score, urinary tract anomalies, and positive urine culture, did not remain significant in multivariable analysis. Full regression results are provided in Table 5. Stone location was correlated with the type of intervention performed: rigid URS was used predominantly for ureteral stones, flexible URS for renal stones, and a combined approach for cases involving both locations. There was no statistically significant association between stone location and postoperative infectious complications. Intraoperative complications were rare and primarily minor—such as mucosal trauma or minor bleeding—and did not show a significant correlation with postoperative infection rates.

**Table 5:** Predictors of postoperative infectious complications: univariable and multivariable logistic regression analysis

Variable	Univariable					Multivariable					
	Estimate	Std. err.	t value	95% CI	*p* value	Estimate	Std. err.	z value	95% CI	*p* value	OR
Age	9.831	3.294	2.294	3.38 to 16.28	0.003						
Sex	0.046	0.089	0.512	− 0.13 to 0.22	0.609						
BMI	− 0.612	1.189	− 0.515	− 2.95 to 1.18	0.607						
ASA score	0.361	0.122	2.964	0.12 to 0.60	0.003						
UT anomalies	− 0.154	0.047	− 3.243	− 0.25 to − 0.06	< 0.001	1.073	0.854	1.257	− 0.60 to 2.75	0.209	2.92
Neurogenic bladder	− 0.238	0.026	− 9.127	− 0.29 to − 0.19	< 0.001	4.467	1.159	3.855	2.20 to 6.74	**< 0.001**	87.2
Urolithiasis	0.31	0.129	2.403	0.06 to 0.56	0.017						
Positive urine culture	0.573	0.136	4.209	0.31 to 0.84	< 0.001	− 0.508	0.331	− 1.533	− 1.16 to 0.14	0.125	0.60
Operative time (min)	3.027	4.35	0.696	− 5.53 to 11.58	0.487						
Time to JJ stent (days)	27.538	6.906	3.987	13.95 to 41.13	< 0.001	− 0.016	0.006	− 2.827	− 0.03 to − 0.01	**< 0.001**	0.98

Univariable and multivariable logistic regression analyses were performed to identify predictors of postoperative infectious complications. Estimates reflect the effect size per unit increase in the corresponding variable. Statistically significant results are shown in bold (*p* < 0.05)

*OR* odds ratio, *CI* confidence interval, *ASA* American Society of Anesthesiologists, *BMI* body mass index (kg/m²), *UT anomalies* urinary tract anomalies

**Fig. 2:**
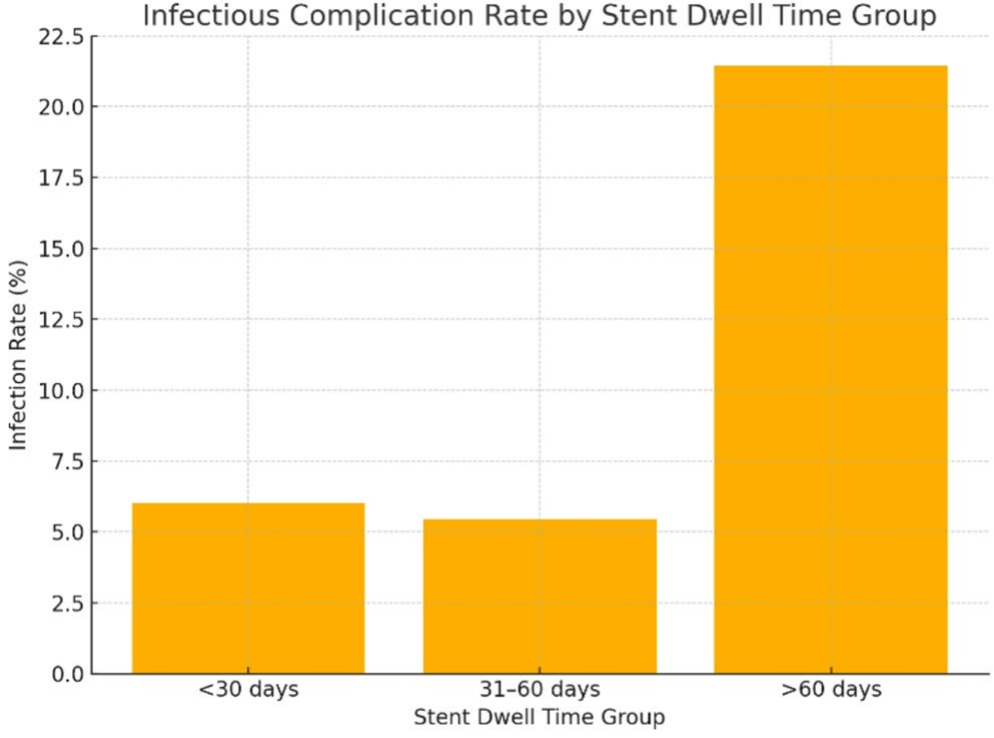
Infectious complication rate by Dwell time

To further evaluate the impact of stent dwell time on infectious complications, patients were stratified into three groups based on stent duration: <30 days, 31–60 days, and > 60 days. Infectious complication occurred in 6.0% of patients with dwell time < 30 days, 5.5% in the 31–60 day group, and 21.4% in those with stents left in place for more than 60 days. A Chi-square test revealed a statistically significant difference in infection rates between these groups (χ² = 9.26, *p* = 0.0098), suggesting that prolonged stent indwelling time, particularly beyond 60 days, is associated with a higher risk of postoperative infectious complications. These findings are summarized in Table 6; Fig. 2.

**Table 6:** Infection rates by dwell time

Groups	Infection count	Total patients	Infection rate (%)
< 30 days	8	133	6.0
31–60 days	7	128	5.5
> 60 days	6	28	21.4

Infection rate represents the proportion of patients in each dwell time group (< 30 days, 31–60 days, > 60 days) who experienced at least one postoperative infectious complication. A Chi-square test was performed to assess differences in infection rates among dwell time groups. The result was statistically significant (χ² = 9.26, *p* = 0.0098), indicating an increased infection risk associated with longer stent dwell times

## Discussion

This study investigated the relationship between preoperative JJ stent dwell time and the risk of infectious complications following URS. Our results demonstrate that prolonged stent duration is significantly associated with increased postoperative infectious failure, with a particularly notable rise in infection risk beyond 60 days of indwelling time.

The findings align with previous reports suggesting that indwelling stents act as a nidus for bacterial colonization, increasing the risk of urinary tract infections and sepsis following endourological procedures. In our cohort, patients with stent dwell times exceeding two months experienced a more than threefold higher rate of infectious complications compared to those with shorter durations. This reinforces earlier studies identifying prolonged stenting as a modifiable risk factor for infection, particularly in high-risk populations [7–10]. However, our study expands upon existing literature by not only confirming this association but also establishing a clinically actionable threshold (>60 days), stratifying dwell time into three meaningful intervals (< 30 days, 31–60 days, and >60 days). This granularity provides a practical framework for surgical planning that has not been emphasized in earlier studies.

Our multivariable analysis further identified neurogenic bladder as an independent predictor of infectious failure. This is consistent with existing literature, where impaired bladder emptying, detrusor dysfunction, and chronic catheterization are well-established contributors to urinary tract infections and postoperative sepsis [11–13]. Neurogenic bladder alters normal voiding dynamics and often necessitates intermittent or indwelling catheterization, both of which facilitate bacterial colonization and biofilm formation [12]. While prior studies have examined infection risk in neurogenic bladder populations, ours is among the few to demonstrate its independent contribution in the context of URS, even after adjusting for other clinical variables such as preoperative bacteriuria or comorbidity scores. This emphasizes that neurogenic dysfunction itself is a dominant risk factor beyond general health status or infection history.

Although other factors such as positive preoperative urine cultures and higher ASA scores showed statistical significance in univariable analyses, their effects were attenuated in multivariable models, highlighting the predominant influence of stent dwell time and neurogenic dysfunction in driving postoperative infectious outcomes. Interestingly, the lack of independent association between positive urine culture and postoperative infection contrasts with prior findings where urine culture was a major predictor [6, 17]. We hypothesize that targeted antibiotic treatment based on culture results in our cohort may have mitigated this risk, and that dwell time served as a stronger mediator of infection than baseline microbial status.

Importantly, our subgroup analysis showed a statistically significant difference in infection rates across dwell time categories (< 30 days, 31–60 days, and >60 days), suggesting that a threshold effect may exist. This observation is supported by prior studies demonstrating that JJ stent indwelling beyond 60 days is associated with higher rates of urinary tract infections, febrile episodes, and complications during URS [14–16]. This stratification may serve as a practical clinical tool to guide surgical planning. Reducing stent dwell times where feasible—ideally limiting preoperative stenting to under two months—may reduce infection-related morbidity and healthcare burden [14, 17].

These findings have direct implications for clinical practice. First, they emphasize the need for streamlined scheduling and prioritization of definitive stone treatment in patients with indwelling JJ stents. Second, they support the development of institutional “fast-track” URS protocols aimed at minimizing unnecessary stent dwell time. Finally, they underscore the importance of careful monitoring and preoperative optimization, particularly in patients with complex urinary tract anatomy or neurogenic bladder.

This study has several limitations. First, its retrospective, single-center design introduces potential selection, information, and observer biases that may have influenced the findings. Although clinical, surgical, and microbiological data were extracted from a well-maintained electronic health record system, retrospective analyses inherently lack control over data completeness and uniformity. Second, some relevant clinical parameters—such as stone burden, stone composition, and precise microbial resistance profiles—were not included in the multivariable analysis. These factors have been previously shown to influence infectious outcomes following URS and could confound the associations observed in this study [18, 19]. That said, we performed a subgroup analysis of stone composition which showed a higher rate of struvite and infectious-related calculi in patients with neurogenic bladder and renal malformations, which may partially explain the heightened infection risk in these groups.

Third, postoperative urine cultures were not systematically performed in all patients but were instead reserved for those exhibiting clinical signs of infection. This practice, while aligned with standard institutional protocols, may have underestimated the true incidence of infectious complications, particularly in cases of asymptomatic bacteriuria. Future prospective studies with standardized microbiological surveillance and routine post-URS cultures could better capture the complete infectious spectrum, including subclinical or delayed infections. In addition, although most patients received cefuroxime in accordance with institutional prophylactic guidelines, the antibiotic regimen was not uniformly standardized across all cases. This variability may have influenced infection outcomes, especially in patients colonized with resistant organisms. Integrating antimicrobial resistance patterns into prophylactic decision-making may improve outcomes, particularly for high-risk subgroups.

Fourth, standardized postoperative imaging to assess stone-free rates (SFR) was lacking. Residual fragments are known infection risk factors, especially in high-risk patients. However, follow-up imaging was inconsistently performed using varied modalities (ultrasound or non-contrast CT), limiting reliable SFR assessment. This represents an area for improvement in future prospective protocols.

Finally, our study population exhibited a sex imbalance, with men comprising 70% of the cohort. This overrepresentation reflects the known epidemiological pattern of higher urolithiasis prevalence in men [20], but it may limit the external validity of our findings, particularly for female patients who may present different risk profiles and stent-related symptomatology [17].

## Conclusion

Prolonged indwelling time of JJ ureteral stents is an independent and clinically significant risk factor for postoperative infectious complications following URS. These findings underscore the importance of minimizing stent dwell time through expedited surgical scheduling and support the development of structured, fast-track care pathways. Implementing such protocols may contribute to reducing infection-related morbidity, improving patient outcomes, and optimizing resource utilization in endourological practice.

## Data Availability

The datasets used and analyzed during the current study are available from the corresponding author on reasonable request.
